# Supplementary material for: Biodegradation in Freshwater: Comparison Between Compostable Plastics and Their Biopolymer Matrices
Source: Polymers (Basel). 2025 Aug 17;17(16):2236. doi: 10.3390/polym17162236 (PMC12390159; doi:10.3390/polym17162236)
Supplement: Supplementary file 1 [file polymers-17-02236-s001.zip › polymers-3803824-supplementary.pdf]

Supporting Information

Biodegradation in Freshwater: Comparison Between Compostable Plastics and Their Biopolymer Matrices

Valerio Bocci [1,2], Martina De Vivo [3], Sara Alfano [3], Simona Rossetti [1], Francesca Di Pippo [1], Loris Pietrelli [4,*] and Andrea Martinelli [3,*]

[1] Water Research Institute, CNR-IRSA, National Research Council, Monterotondo, 00015 Rome, Italy

[2] PhD Program in Evolutionary Biology and Ecology, Department of Biology, University of Rome 'Tor Vergata', 00133 Rome, Italy

[3] Department of Chemistry, Sapienza University of Rome, 00185 Rome, Italy

[4] Legambiente, Scientific Committee, 00199 Rome, Italy

* Correspondence: l.pietrelli@legambiente.it (L.P.); andrea.martinelli@uniroma1.it (A.M.);

Tel.: +39-3385411127 (L.P.); +39-0649913950 (A.M.)

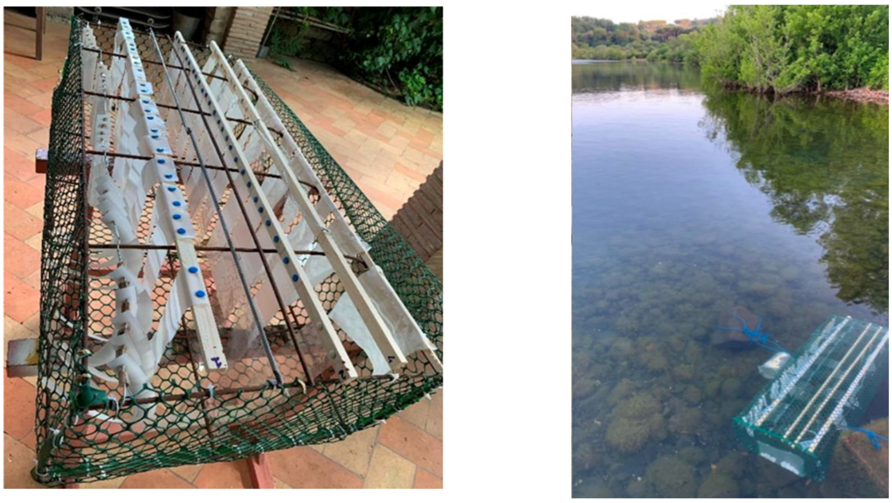

Figure S1. Images of the cage with the samples and the site of Bracciano lake where it is immersed

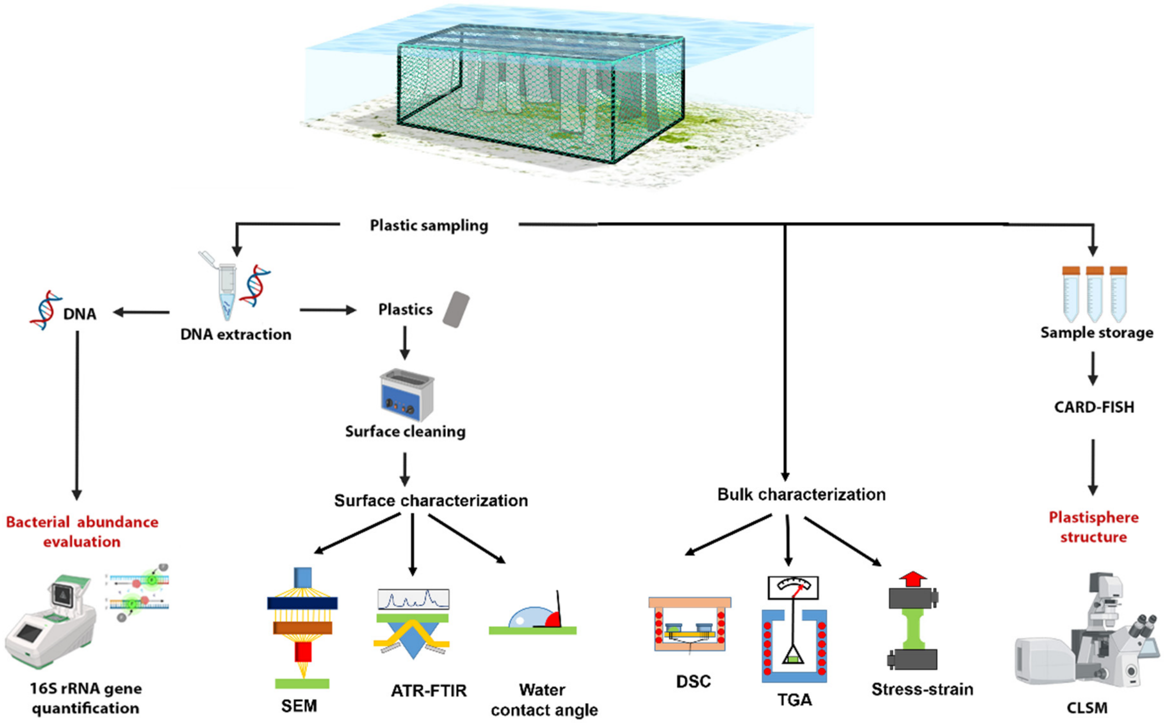

Figure S2. Scheme of the experimental set up

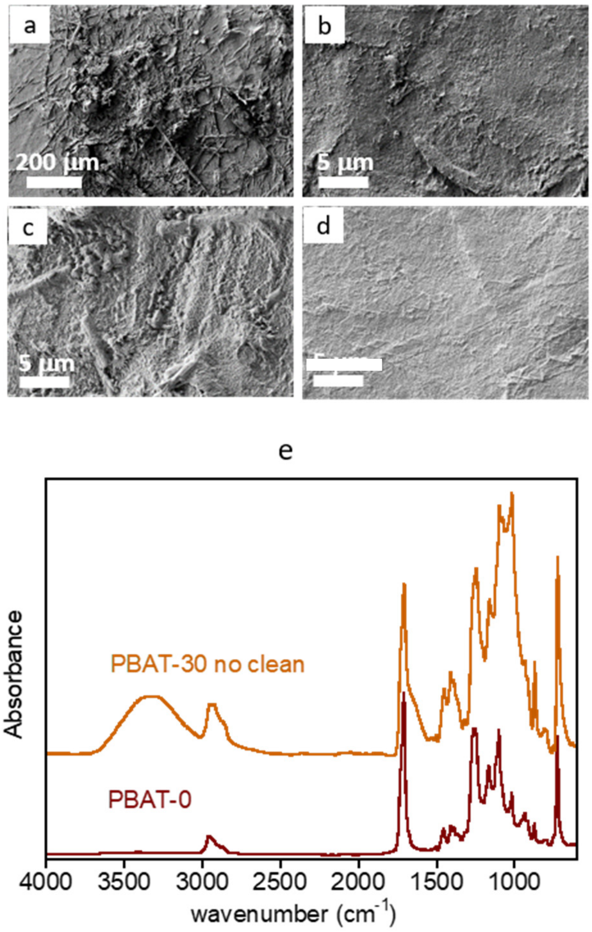

Figure S3. SEM images of PBAT-30 before (a) and after cleaning wit Method 1 (b), Method 2 (c) and Method 1 followed by Method 2 (d). (e) Comparison of the ATR-FTIR spectra of the sample PBAT-30 acquired before (PBAT-30 not cleaned) and after the double cleaning procedure (PBAT-30 cleaned).

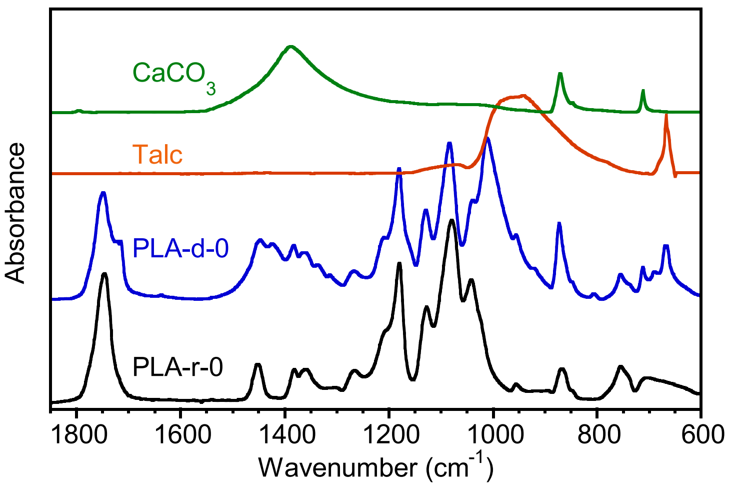

Figure S4. PLA-d and PLA-r surface characterization. FTIR-ATR spectra of pure PLA (PLA-r-0), PLA based dish (PLA-d-0) recorded before the immersion in freshwater as well as of calcium carbonate ($CaCO_3$) and talc, found as filler in plastic dish

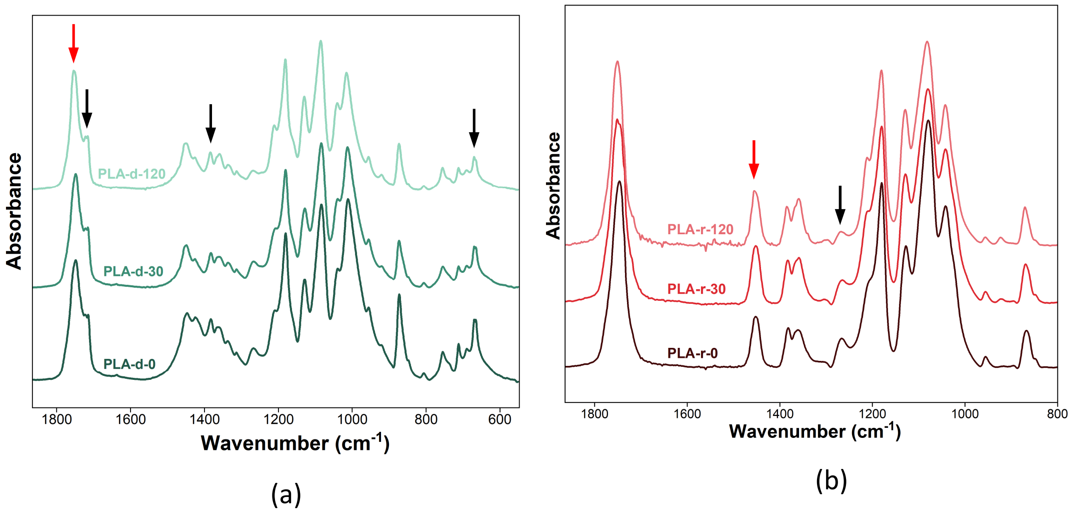

Figure S5. (**a**) ATR-FTIR spectra of PLA-d sample immersed for 0, 30 and 120 days. (**b**) ATR-FTIR spectra of PLA-r sample immersed for 0, 30 and 120 days. Red arrows indicate the normalization bands, black arrows the bands of the PLA-d fillers (**a**) and the band of the PLA-r amorphous phase (**b**)

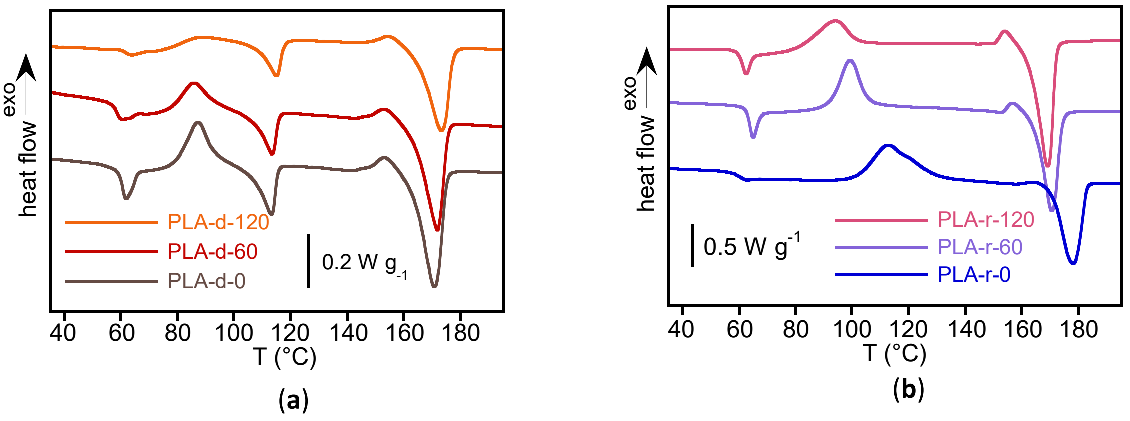

Figure S6. (**a**) DSC heating curves of PLA-r samples immersed in freshwater for 0, 60 and 120 d. (**b**) DSC heating curves of PLA-d samples immersed in freshwater for 0, 60 and 120 d

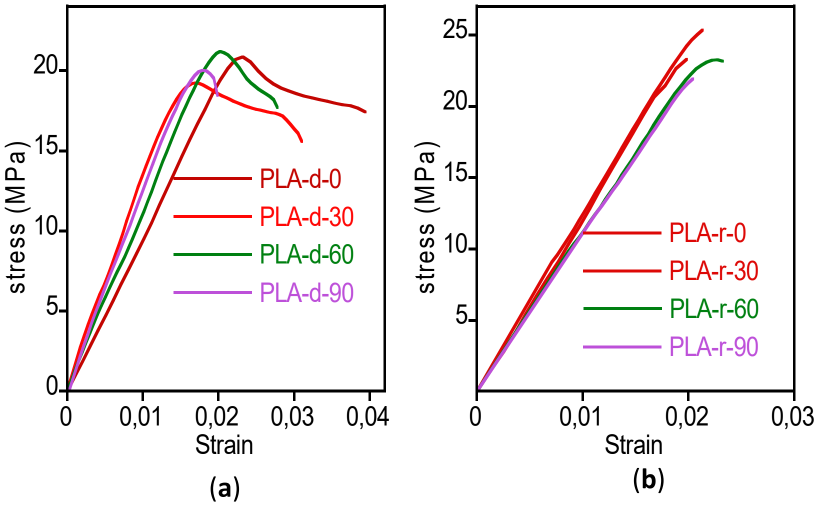

Figure S7. Mechanical characterization. Selected strass-strain curves of PLA-d (**a**) and PLA-r (**b**) samples immerse for 0, 30, 60 and 90 days

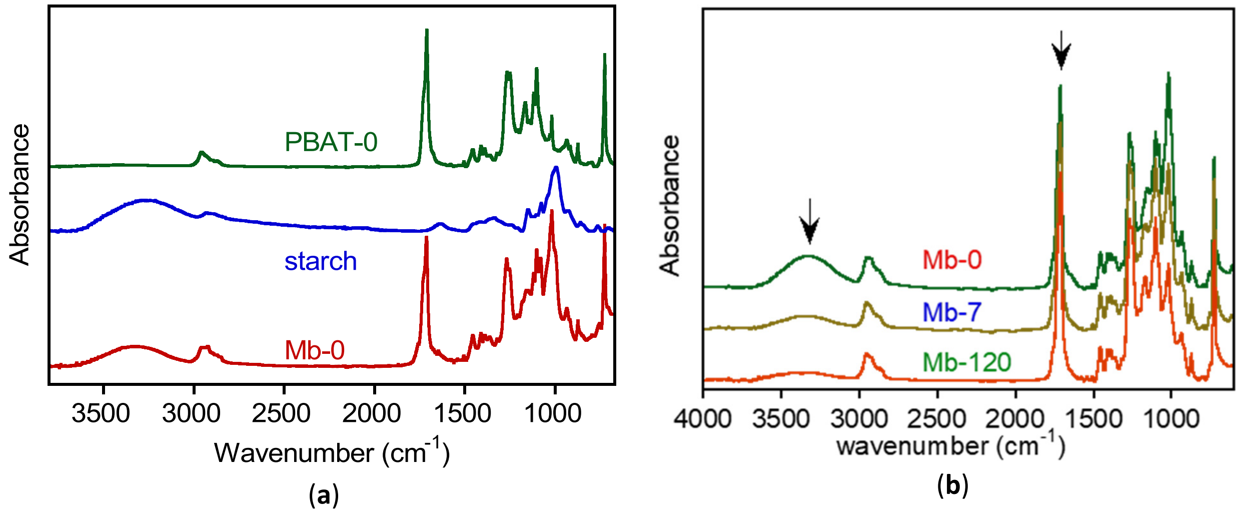

Figure S8. (**a**) ATR-FTIR spectra of Mb, starch and PBAT. (**b**)Mb spectra recorded after 0, 7 and 120 days. The arrows indicate the band centered at 3340 cm^{-1}, assigned to O-H stretching of the polysaccharide, and the band at 1711 cm^{-1} of PBAT C=O stretching whose intensity was used to normalize the spectra.

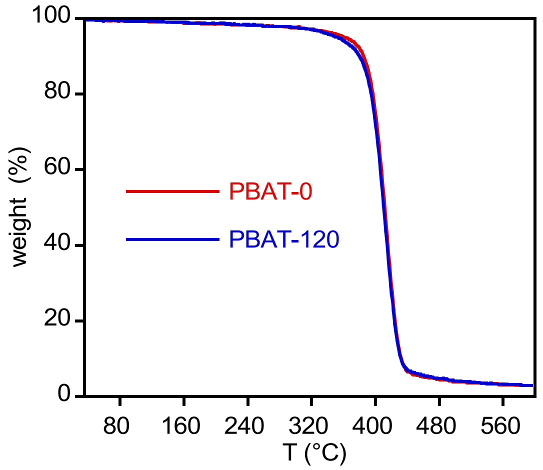

Figure S9 Thermogravimetric analysis. TGA curves of PBAT recorded after 0 and 120 days of immersion.

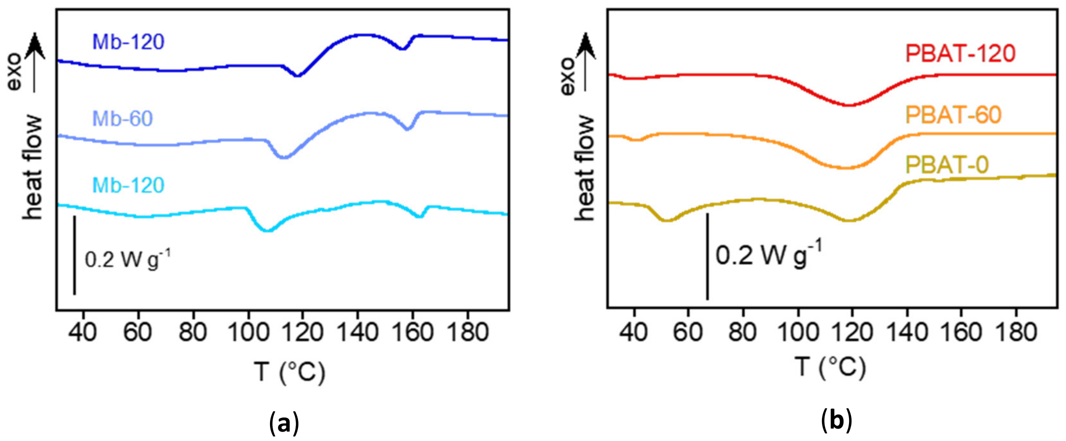

Figure S10. DSC heating curves of Mb (**a**) and PBAT (**b**) acquired after 0, 60 and 120 days of immersion.

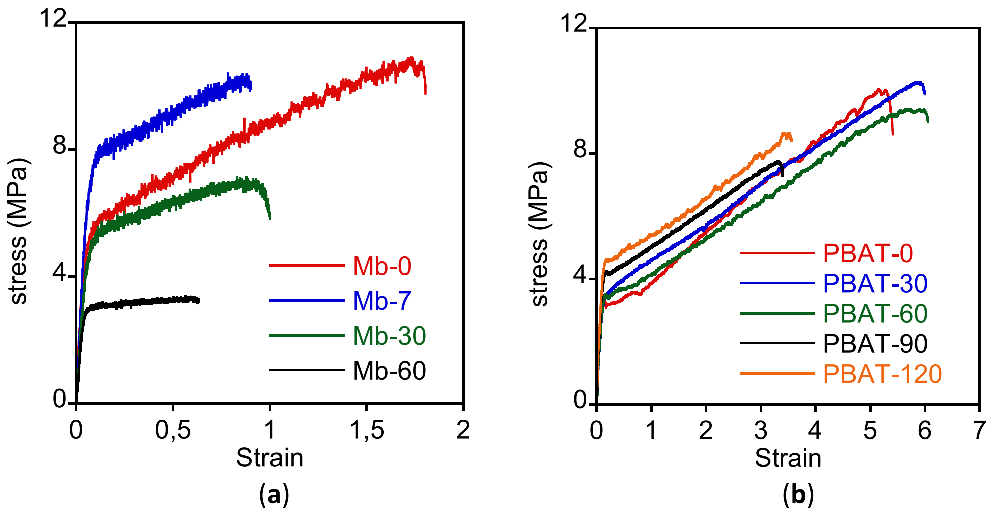

Figure S11. Mb and PBAT mechanical characterization. Selected strass-strain curves of Mb (**a**) and PBAT (**b**)

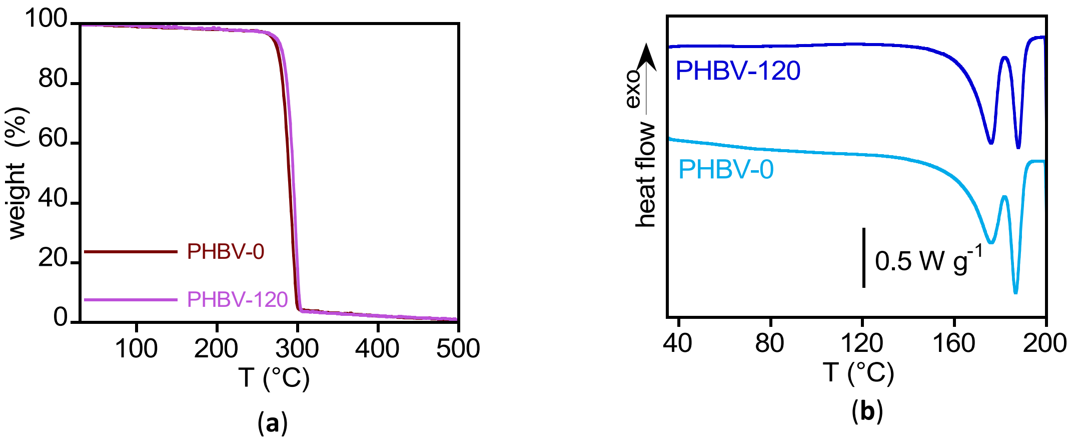

Figure S12. (**a**) TGA curves of PHBV recorded after 0 and 120 days of immersion. (**b**) DSC thermograms of PHBV acquired after 0 and 120 days of immersion.

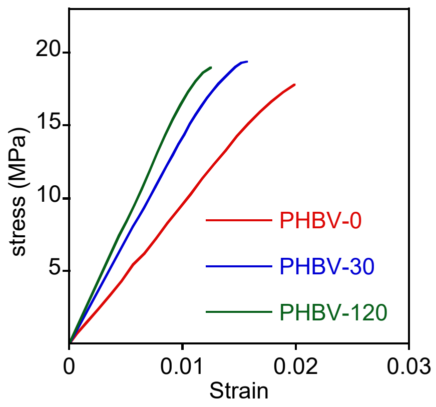

Figure S13. PHBV mechanical characterization. Selected strass-strain curves of PHBV samples immerse for different time periods

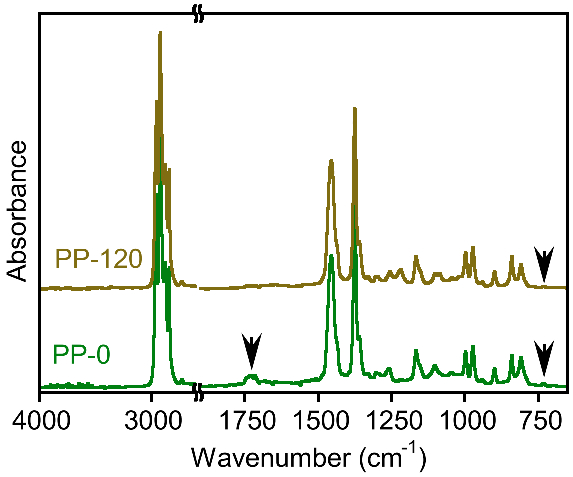

Figure S14. ATR-FTIR spectra of pristine PP sample (PP-0) and immersed for 120 days (PP-120). The arrows highlight the weak absorptions of polyethylene (730 cm^{-1}) and of the additive (1725 cm^{-1}).

Table S1. 16S rRNA gene quantification (GC cm^{-2}).

	Days			
	30	60	90	120
**PBAT**	(1.4±0.1) $\times10^8$	(1.97±0.04) $\times10^9$	(1.8±0.4) $\times10^9$	(1.1±0.1) $\times10^7$
**Mb**	(5.2±0.6) $\times10^9$	(2.7±0.6) $\times10^9$	(2.2±0.1) $\times10^9$	(4.4±0.4) $\times10^8$
**PLA-r**	(2.5±0.1) $\times10^8$	(2.8±0.1) $\times10^8$	(7.30±0.2) $\times10^8$	(5.2±0.4) $\times10^6$
**PLA-d**	(2.3±0.2) $\times10^6$	(6.1±0.4) $\times10^8$	(4.27±0.06) $\times10^8$	(5.0±0.2) $\times10^6$
**PHBV**	(1.2±0.1) $\times10^8$	(1.93±0.02) $\times10^9$	(3.3±0.2) $\times10^8$	(2.3±0.1) $\times10^6$
**PP**	(3.8±0.1) $\times10^8$	4.10±0.9) $\times10^8$	(3.9±0.1) $\times10^8$	(3.62±0.06) $\times10^7$

Standard deviation values are based on three experimental replicates

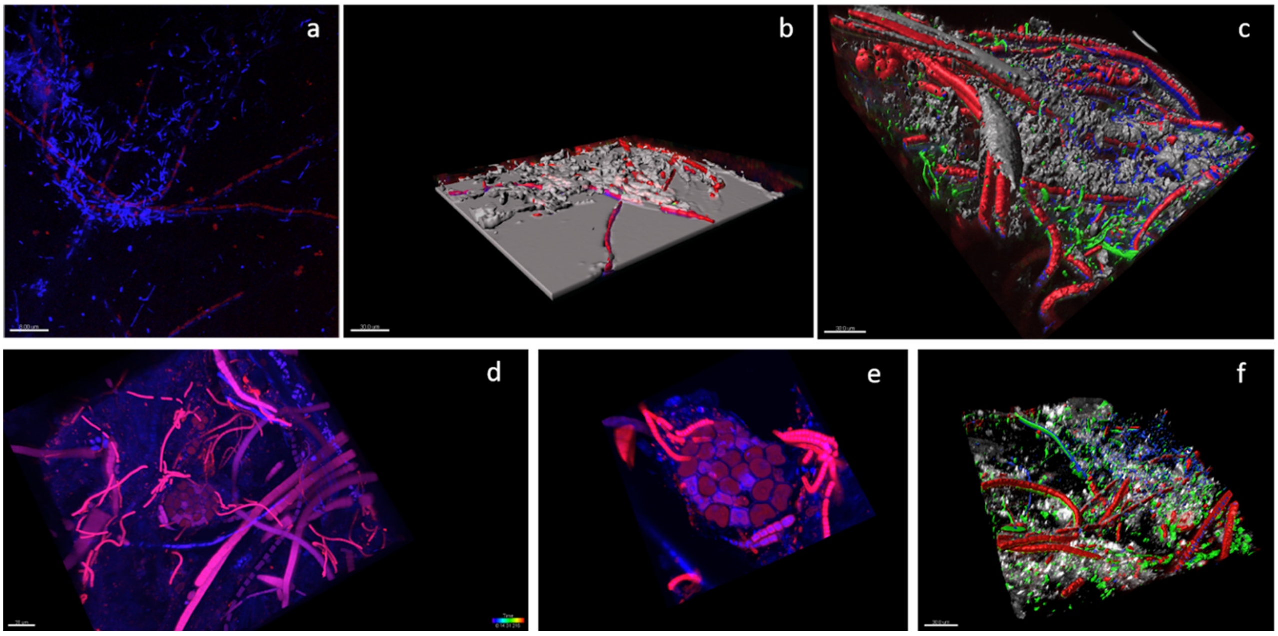

Figure S15. CLSM micrographs of the plastisphere grown on different materials after exposure to lake waters. Total viable cells stained with DAPI (blue signal) in the Mb-associated biofilms after 7 days of immersion (red signal is the autofluorescence of chlorophyll) (a). 3-D reconstruction of biofilm communities from PHBV (b) and Mb (c), hosting microbial cells and filaments. 3-D reconstruction of biofilm communities on Mb (d, e) and PLA (f) during the late stages of microbial development, when the plastisphere was composed of both bacterial (purple signal) and eukaryotic phototrophs (red signal).
